# Immunocytochemical studies reveal novel neural structures in nemertean pilidium larvae and provide evidence for incorporation of larval components into the juvenile nervous system

**DOI:** 10.1186/1742-9994-10-31

**Published:** 2013-05-23

**Authors:** Sabine Hindinger, Thomas Schwaha, Andreas Wanninger

**Affiliations:** 1 Faculty of Life Sciences, Department of Integrative Zoology, University of Vienna, Althanstr. 14, Vienna, 1090, Austria

**Keywords:** Evolution, Neurogenesis, Development, Pilidium, Lophotrochozoa, Confocal microscopy, Ribbon worm

## Abstract

**Introduction:**

Nemertea is one of the least studied lophotrochozoan phyla concerning neurogenesis. The sparse data available do not unambiguously allow for answering questions with respect to the neural groundplan of the phylum or the fate of larval neural structures during metamorphosis. In order to contribute to this issue, we studied neurotransmitter distribution during development of the pilidiophoran *Lineus albocinctus* Verrill, 1900.

**Results:**

Two serotonin-like immunoreactive (lir) neurons are present in the anterior part of the apical plate. They send numerous processes into the four lobes of the pilidium larva, where they form a complex subepithelial nerve net. All four larval lobes are underlain by a marginal neurite bundle, which is associated with numerous serotonin-lir monociliated perikarya. A serotonin-lir oral nerve ring encircles the stomach sphincter and is associated with few serotonin-lir conical cells. Two suboral neurites descend from the oral nerve ring and merge with the marginal neurite bundle. The oral nerve ring and the suboral neurites contain the mollusk-specific VD1/RPD2 α-neuropeptide. The lateral lobes of the larva have three and the anterior and the posterior lobes two VD1/RPD2-lir marginal neurite bundles. The lobar FMRFamide-lir plexus of *Lineus albocinctus* is much more complex than previously described for any pilidium larva. It includes a circumesophageal neurite that descends along each side of the larval esophagus and together with the inner marginal neurite bundle gives rise to the lobar plexus of the lateral lobes. An outer FMRFamide-lir marginal neurite bundle with numerous associated FMRFamide-lir marginal sensory cells surrounds all four lobes. FMRFamide-lir structures are absent in the larval apical region. The oral nerve ring and the two suboral serotonin-lir neurites are incorporated into the juvenile nervous system.

**Conclusion:**

Our study confirms the presence of serotonin-lir components in the apical region of the pilidium larva of *Lineus albocinctus* and thus contradicts an earlier study on the same species. We show that the nervous system of pilidium larvae, especially the FMRFamide-lir components, is much more complex than previously assumed. The presence of the VD1/RPD2-α-neuropeptide indicates that this compound may have been part of the lophotrochozoan neural groundplan.

## Introduction

Adult Nemertea, also known as ribbon worms, are benthic predators and are predominantly found in marine habitats, but freshwater and terrestrial species also exist [1,2]. They are bilaterally symmetric, unsegmented and dorsoventrally flattened. One apomorphic character for the phylum is the eversible and sometimes stylet-bearing epidermal proboscis [1,3]. It lies within a fluid-filled cavity of mesodermal origin, the so-called rhynchocoel. Unlike in many other protostome invertebrates, the nemertean brain encircles the proboscis and the two frontal blood vessels, but not the foregut [1,4,5].

**Figure 1:**
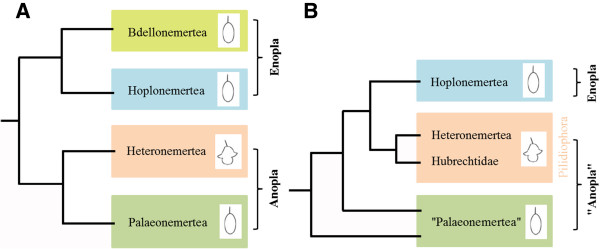
**Suggested nemertean interrelationships.** (**A**) Traditional nemertean phylogeny based on adult morphology, modified after [14]. The phylum is divided into two sub-taxa: Anopla and Enopla. Anopla comprises Palaeonemertea and Heteronemertea. Enopla comprises Hoplonemertea and Bdellonemertea. (**B**) Nemertean interrelationships based on EST data, modified after [15]. “Palaeonemertea” is a paraphyletic assemblage where only some of its representatives constitute a basal offshoot within Nemertea. The Pilidiophora contain the Heteronemertea and the Hubrechtidae and form a monophyletic clade whose members develop via a pilidium larva. The sistergroup to the Pilidiophora is the monophyletic Hoplonemertea. The Hoplonemertea, which in this phylogeny are a synonym for Enopla, include Bdellonemertea. Similar to the “Palaeonemertea”, its representatives develop via a planuliform larva.

Nemertea display two major developmental modes that strikingly differ from each other. Palaeonemertea, which are considered to be paraphyletic, and Hoplonemertea exhibit a planuliform larva, while many species of the Pilidiophora have a long-lived planktotrophic pilidium larva (Figure 1A, B; [6]). Anteriorly, this latter larval type exhibits a pointed episphere with an apical plate. It is formed by columnar ciliated epidermal cells which give rise to the apical tuft, a characteristic feature of many trochozoan larval apical organs [7,8]. The episphere is surrounded by four lobes; an anterior, a posterior and two lateral lobes. Short cilia cover the larval epidermis, while a band of longer cilia runs along the margin of the four lobes. Between the two lateral lobes the opening of the esophagus, the so-called vestibule, is situated [9]. It terminates in a blind stomach characterized by a thick epithelium. Esophagus and stomach are separated by a muscular sphincter [9]. In the fully differentiated larva a set of three paired epidermal imaginal discs and two unpaired, probably mesenchymal, imaginal discs are formed [9-11]. These imaginal discs fuse, grow over the larval stomach, and eventually form the juvenile worm, which comes to lie within the larval episphere. During a so-called catastrophic metamorphosis, the juvenile worm emerges from the larva and swallows the remaining larval tissue [4,9,12,13].

The development of the nemertean larval nervous system has been studied by various methods in the hoplonemertean *Quasitetrastemma stimpsoni* and in different species of Pilidiophora. Transmission electron microscopy (TEM) investigations revealed the presence of a marginal nerve that runs along the four larval lobes in undetermined pilidium larvae and in the larva of *Lineus albocinctus*[8,16]. In addition, two cell types, each with a single cilium surrounded by a microvilli collar, are present in an undetermined pilidium larva. These cells are always associated with the marginal nerve [8]. In an undetermined pilidium and in *Lineus albocinctus* an oral nerve ring encircles the sphincter between esophagus and stomach. Two suboral nerves descend from the oral nerve in both species. They merge with the marginal nerve at the transition between the posterior and the lateral lobes in an undetermined pilidium [8]. Ultrastructural investigations showed that in the larva of *Lineus albocinctus* the suboral nerve splits into an anterior and a posterior portion; both merge with the marginal nerve at the transition between the lobes. An additional pair of so-called “lateral helmet nerves” descends from the anterior part of the anterior lobe to the junction of the anterior and the lateral lobes, where it merges with the marginal nerve [16]. Interestingly, no neurons associated with the apical plate could hitherto be found by TEM analysis in any pilidiophoran larva [8,16].

Immunocytochemical investigations of the pilidiophorans *Lineus albocinctus* and *Micrura alaskensis* revealed a serotonin-lir marginal nerve that underlies the ciliary band of the four larval lobes. An extensive subepithelial serotonin-lir nerve net with numerous interconnecting serotonin-lir neurons and a serotonin-lir oral nerve ring is present in the larvae of *Lineus albocinctus* and *Micrura alaskensis*, but apical serotonin-lir structures were only found in the pilidium larva of *Micrura alaskensis*[9,16]. Juveniles of both species show a pair of serotonin-lir lateral nerve cords that emerges from the cephalic discs and runs ventro-laterally. In addition, several longitudinal serotonin-lir proboscis neurites are present [9,16]. To date, data on FMRFamide-lir structures are available only for one pilidiophoran species, namely *Lineus albocinctus*, where one to four FMRFamide-lir cells were found in the larval episphere. They send their processes to the anterior region of the apical plate and to the junction between the anterior and the lateral lobes. In the juvenile of *Lineus albocinctus* no FMRFamide-lir structures were found [16].

Despite these data on larval neuroanatomy, development of immunoreactive compounds in Nemertea, especially Pilidiophora, remains largely unknown, as is the fate of the larval nervous system and the question as to what degree larval components are incorporated into the juvenile neural bodyplan. In order to address these issues and to provide novel data on the neural architecture of nemertean pilidium larvae, we analyzed neurogenesis of *Lineus albocinctus* using antibodies directed against the common neurotransmitters serotonin and FMRFamide as well as the VD1/RPD2 α-neuropeptide, which was originally described from the mud snail, *Lymnaea stagnalis*[17], but seems to be present in non-molluscan invertebrates as well [17-19].

## Results

### Terminology

Several neuroanatomical terms, such as “nerve” and “nerve cord”, are used in different ways for various invertebrate taxa without any consensus on their exact definitions. A recently published glossary on neuroanatomical terminology defines a “nerve” as a structure of condensed axons free of cell bodies [20]. Whole-mount immunocytochemical methods, however, usually neither allow for *in toto* visualization of entire nerves (including their cell bodies, entity of axons and dendrites) nor for a clear distinction between axons and dendrites. Therefore, terms such as “nerve” and “nerve cord” are only used herein if they were unambiguously identified by transmission electron microscopy (TEM). In accordance with a recent paper [20] we use “neurite” and “neurite bundle” if the data were generated by immunocytochemistry only.

### Development of serotonin-lir structures from early larva to the juvenile worm

**Figure 2:**
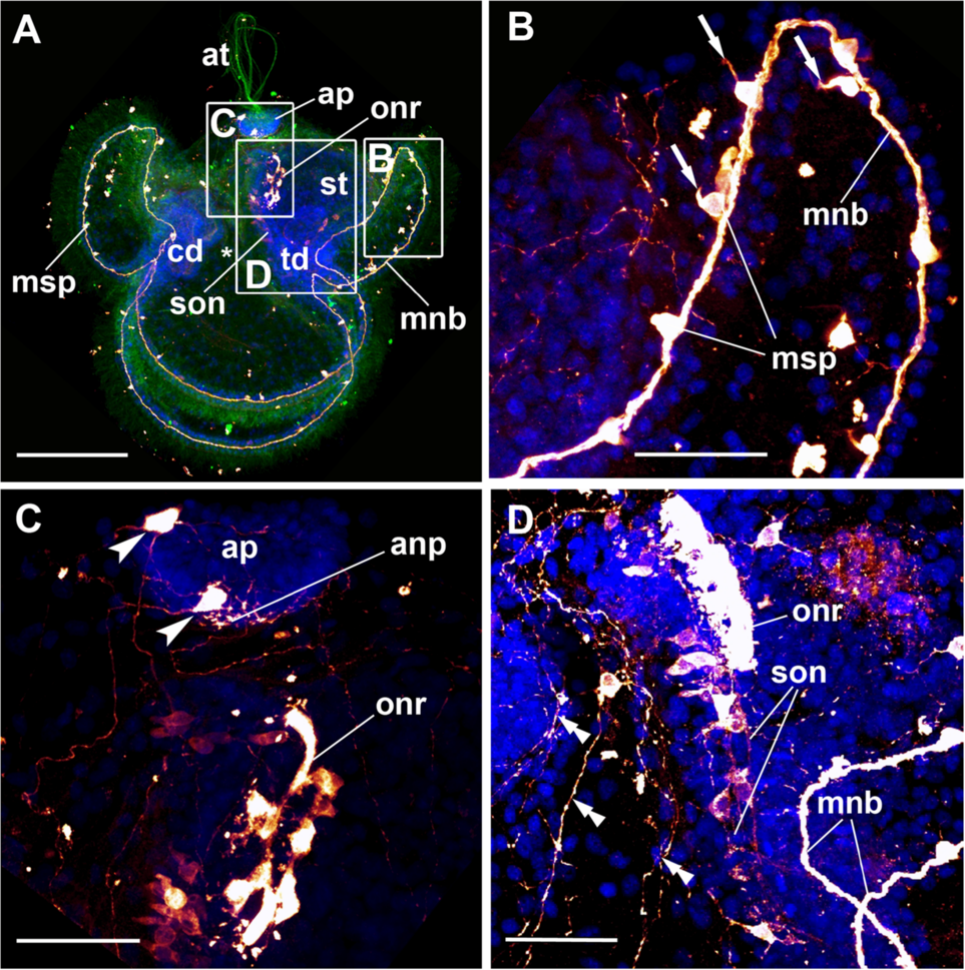
**Serotonin-lir neural structures in an early *Lineus albocinctus *larva.** Serotonin-lir is in graded scales of dark red to bright yellow, cell nuclei are in blue, tubulin in green. Apical faces upwards. All images are maximum projections of scans through the entire specimen. Boxed areas in **A** indicate details of **B**, **C** and **D**. Image **D** is from a different specimen. (**A**) Larva with early *anlagen* of cephalic and trunk discs. The apical plate gives rise to the apical ciliary tuft and shows two serotonin-lir neurons. An oral nerve ring encircles the stomach and gives rise to two serotonin-lir suboral neurites, which merge with the marginal neurite bundle at the junction of the posterior and the lateral lobes. Scale bar 100 μm. (**B**) Detailed view of the marginal neurite bundle of the posterior lobe. Marginal serotonin-lir perikarya bear a single cilium each. Scale bar 30 μm. (**C**) Detail of the apical plate region and the oral nerve ring. Two serotonin-lir neurons of the apical plate send their processes to the four lobes and form an apical neurite plexus. Scale bar 30 μm. (**D**) Detail of the oral nerve ring and the suboral neurites. The suboral neurite are always associated with conical serotonin-lir cells. Scale bar 30 μm. Abbreviations: *, mouth; anp, apical neurite plexus; ap, apical plate; at, apical ciliary tuft; cd, cephalic disc; mnb, marginal neurite bundle; msp, marginal serotonin-lir perikaryon; onr, oral nerve ring; son, suboral neurite; st, stomach; td, trunk disc; arrows, single cilium; arrowheads, serotonin-lir neurons; double arrowheads, lateral processes.

**Figure 3:**
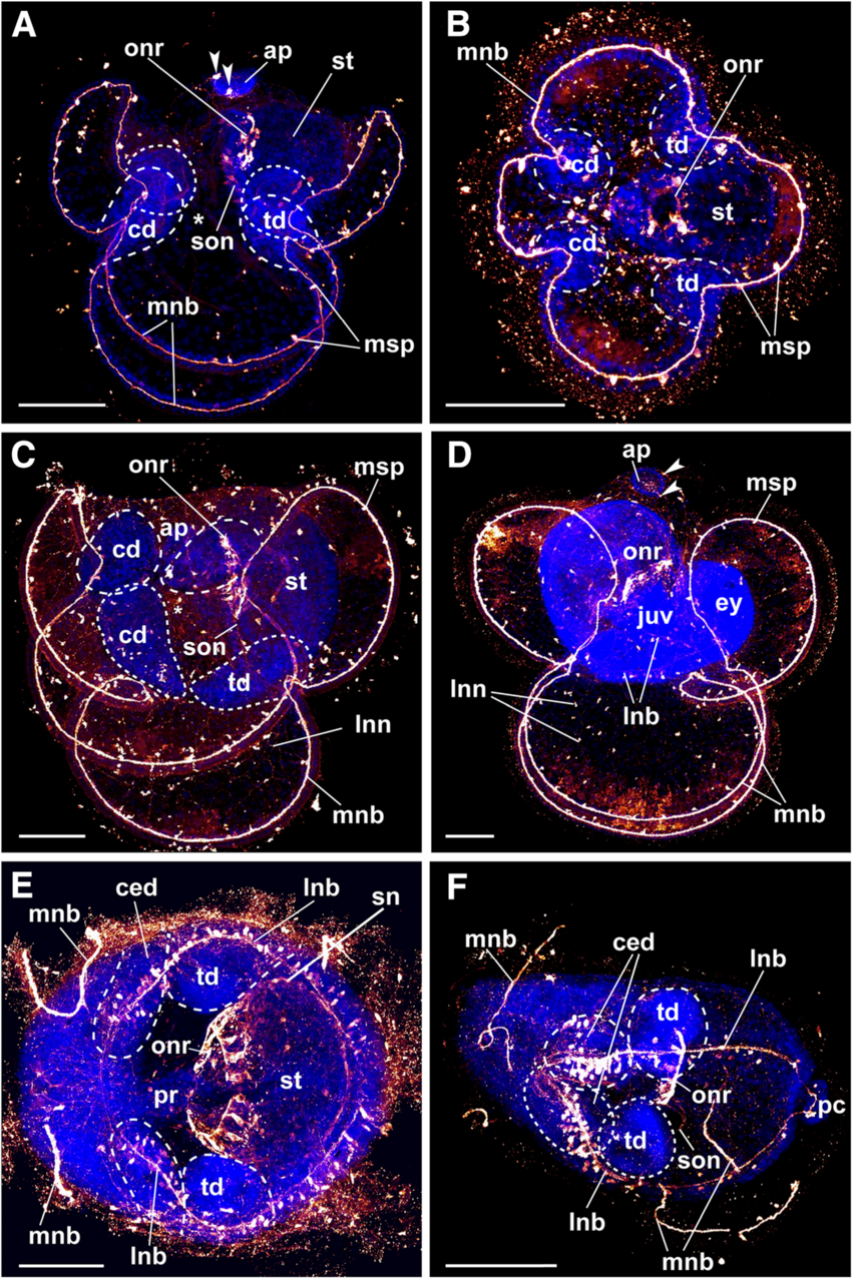
**Serotonin-lir neurogenesis in the pilidium larva and the juvenile of *Lineus albocinctus*.** Serotonin-lir is shown in graded scales of dark red to bright yellow, cell nuclei are in blue. Apical of the larvae faces upwards in **A**, **C** and **D**. **B** is an abapical view. Larva with incorporated trunk disc stage in **A** and **B**. Anterior of the dissected juvenile is to the left in **E** and **D**. All images are maximum projections of scans through the entire specimen. White dotted lines encircle the imaginal discs of the developing juvenile. (**A**) Same specimen as in 2A. The apical plate shows two serotonin-lir neural cell bodies. A serotonin-lir oral nerve ring encircles the stomach and sends two suboral neurites towards the transition between the posterior and the lateral lobes. (**B**) The marginal neurite bundle underlies all four lobes. It is associated with marginal serotonin-lir perikarya. (**C**) The processes of the apical neurons form a complex lobar nerve net with numerous interconnected multipolar interneurons. (**D**) All imaginal discs and *anlagen* have fused and have formed a juvenile worm. The oral nerve ring and the two suboral neurites have been incorporated into the juvenile body. (**E**) Dissected juvenile. Two lateral neurite bundles emerge from the fused cephalic discs and run along the ventro-lateral side of the juvenile. (**F**) Dissected juvenile. The lateral neurite bundles show a high density of associated serotonin-lir cells within the cerebral organ discs. All scale bars equal 100 μm. Abbreviations: *, mouth; ap, apical plate; cd, cephalic disc; ced, cerebral organ disc; ey, eye; juv, juvenile worm; lnb, lateral neurite bundle; lnn, lobar nerve net; mnb, marginal neurite bundle; msp, marginal serotonin-lir perikarya; onr, oral nerve ring; pc, posterior cirrus; pr, proboscis *anlage*; sn, stomach neurite; son, suboral neurite; st, stomach; td, trunk disc; arrowheads, apical neuron.

**Figure 4:**
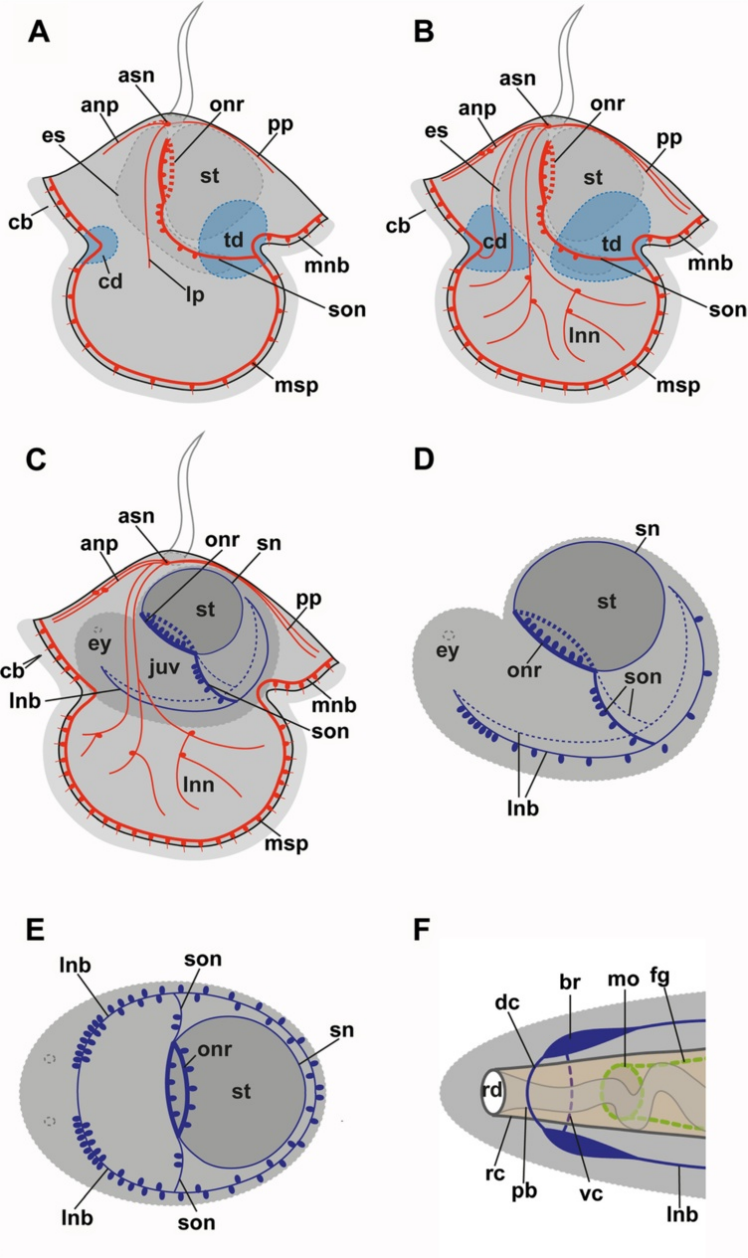
**Line drawings illustrating development of the serotonin-lir nervous system in *Lineus albocinctus*.** Larval serotonin-lir components in red, juvenile serotonin-lir structures in blue except for **F**, which depicts the overall adult neuroanatomy. Apical faces upwards in **A**, **B**, **C**. Anterior is to the left in **D**, **E**, **F**. (**A**) Larva with incorporated trunk disc stage. Two apical sensory neurons send their processes to the four lobes. The oral nerve ring encircles the stomach and gives rise to two suboral neurites. Size is approximately 400 μm. (**B**) Larva with almost fused cephalic and trunk discs. Compared to the specimen in **A**, a higher number of serotonin-lir processes, which originate from the two apical neurons and project into all four lobes, is present. Size is approximately 470 μm. (**C**) Juvenile worm inside the larval episphere. The oral nerve ring and the suboral neurites (dark blue) have been incorporated into the juvenile. Two lateral neurite bundles appear on the ventro-lateral sides. Size is approximately 830 μm. (**D**) Isolated juvenile, lateral left view. The two suboral neurites merge with the lateral neurite bundles. Size is approximately 380 μm. (**E**) Juvenile, dorsal view. Two lateral neurite bundles run along the ventro-lateral sides. Size is approximately 430 μm. (**F**) Anterior region of an adult “Anopla” after [1]. The brain encircles the rhynchocoel but not the foregut. Abbreviations: anp, anterior neurite plexus; asn, apical sensory neuron; br, brain; cb, ciliary band; cd, cephalic disc; dc, dorsal commissure; es, esophagus; ey, eye; fg, foregut; juv, juvenile worm; lnb, lateral neurite bundle; lnn, lobar nerve net; lp, lateral plexus; mnb, marginal neurite bundle; mo, mouth opening; msp, marginal serotonin-lir perikaryon; onr, oral nerve ring; pb, proboscis; pp, posterior plexus; rc, rhynchocoel; rd, rhynchodaeum; sn, stomach neurite; son, suboral neurite; st, stomach; td, trunk disc; vc, ventral commissure.

The earliest larval stages investigated show the cephalic discs of the juvenile as small pouches at the transition between the anterior and the lateral lobes, as well as the trunk discs, which invaginate at the transition of the posterior and the lateral lobes (Figures 2A, 3A, B and 4A). These stages are addressed as “larvae with incorporated trunk disc stages” in the following [9]. The cerebral organ discs, the proboscis *anlage* and the dorsal *anlage* have not developed at this stage. The prominent apical plate is present and shows two serotonin-lir cell bodies in the anterior part of the apical plate, one cell on the left and one on the right side (Figures 2A, C and 3A). These apical neurons send processes to the anterior, posterior and the lateral lobes, where they branch and contribute to the lobar neurite plexus (Figure 4B, C). Beneath the apical plate, the processes of the apical neurons form a complex apical neurite plexus (Figure 2C). Several of these processes that originate from the apical neurons merge with the marginal neurite bundle, which is a serotonin-lir neurite bundle that runs along the four larval lobes. Serotonin-lir monociliated perikarya are always associated with the marginal neurite bundle. Their number increases during development (Figure 4A-C). At the junction between the esophagus and the stomach a prominent serotonin-lir nerve ring with several associated conical cell bodies is present and encircles the sphincter region (Figure 2A, C, D and 3A-D). Two suboral neurites originate from the oral nerve ring and descend towards the transition between the posterior and the lateral lobes, where they merge with the marginal neurite bundle (Figures 2D, 3A, C and 4A). The suboral neurites as well as the oral nerve ring are connected to numerous serotonin-lir cell bodies (Figures 2C, D and 4A). More advanced stages show almost fused cephalic discs and more developed trunk discs (Figures 3C and 4B). The processes that emanate from the apical neurons have increased in number at this stage (Figure 4B). In addition, a distinct subepithelial nerve net is present in all four lobes (Figures 3C and 4B, C). During larval development these nerve nets increase in complexity, as does the number of multipolar interneurons, which interconnect the individual neurites (Figure 3C, D). The number of serotonin-lir cells associated with the marginal neurite bundle is higher in this developmental stage than in larvae at the incorporated trunk discs stage. The oral nerve ring appears more prominent and shows an increase in associated conical cell bodies (Figure 3A, C). Later in development, all three pairs of imaginal discs as well as the proboscis *anlage* and the dorsal *anlage* of the juvenile fuse and form the juvenile worm that lies in the episphere of the larva (Figures 3D and 4C). The anterior-posterior axis of the juvenile is almost perpendicular to that of the larva (Figures 3D and 4C). The imaginal discs grow over the larval digestive tract and incorporate it into the juvenile body, resulting in a shared digestive tract of the juvenile and the larva (Figures 3D and 4C). The larval serotonin-lir nervous system in this stage differs from that of earlier larvae (Figures 3C, D and 4C). The two apical neurons and their processes, which form the complex nerve net, are still present. The larva exhibits the marginal neurite bundle and its associated monociliated serotonin-lir perikarya. Two lateral neurite bundles with several associated conical cell bodies are now present along the ventro-lateral side of the juvenile worm (Figure 4C). Interestingly, the oral nerve ring and the suboral neurites are now visible within the juvenile body, whereby the suboral neurites begin to merge with the juvenile lateral neurite bundle at the site of origin of the trunk discs (Figure 3F).

Dissected juveniles, one with almost fused discs (“torus stage”, see [9]) and one with the discs and *anlagen* that have entirely fused, show, that the oral nerve ring and the suboral neurites are incorporated into the juvenile nervous system (Figures 3E, F and 4C-E). The suboral neurites now descend into the trunk disc portion of the lateral neurite bundles of the juvenile and are no longer connected to the larval marginal neurite bundle (Figures 3F and 4C-E). Additionally, several neurites that originate from the oral nerve ring and run around the stomach along the anterior-posterior axis of the juvenile are formed (Figures 3E and 4C-E). Two prominent lateral neurite bundles are present along the ventral side of the juvenile worm (Figures 3D-F and 4C-E). They emerge from the posterior part of the already fused cephalic discs, transverse the cerebral organ disc region, continue laterally where they transverse the trunk discs, and project into the very posterior region of the juvenile (Figure 3E, F). The cephalic discs region is traversed by numerous serotonin-lir neurites connected to the lateral neurite bundles (Figure 3E, F). However, this region shows no serotonin-lir cell bodies associated with the lateral neurite bundle. The proboscis *anlage* exhibits several serotonin-lir neurites along its anterior-posterior axis. In the region of the cerebral organ the lateral neurite bundles express a high density of associated serotonin-lir cells (Figure 3F). Along the trunk discs and in the posterior region of the juvenile such cells are also present, but are lower in number (Figures 3F and 4D, E; for comparison with the adult neural condition see Figure 4F).

### Development of FMRFamide-lir structures from the early larva to the juvenile worm

**Figure 5:**
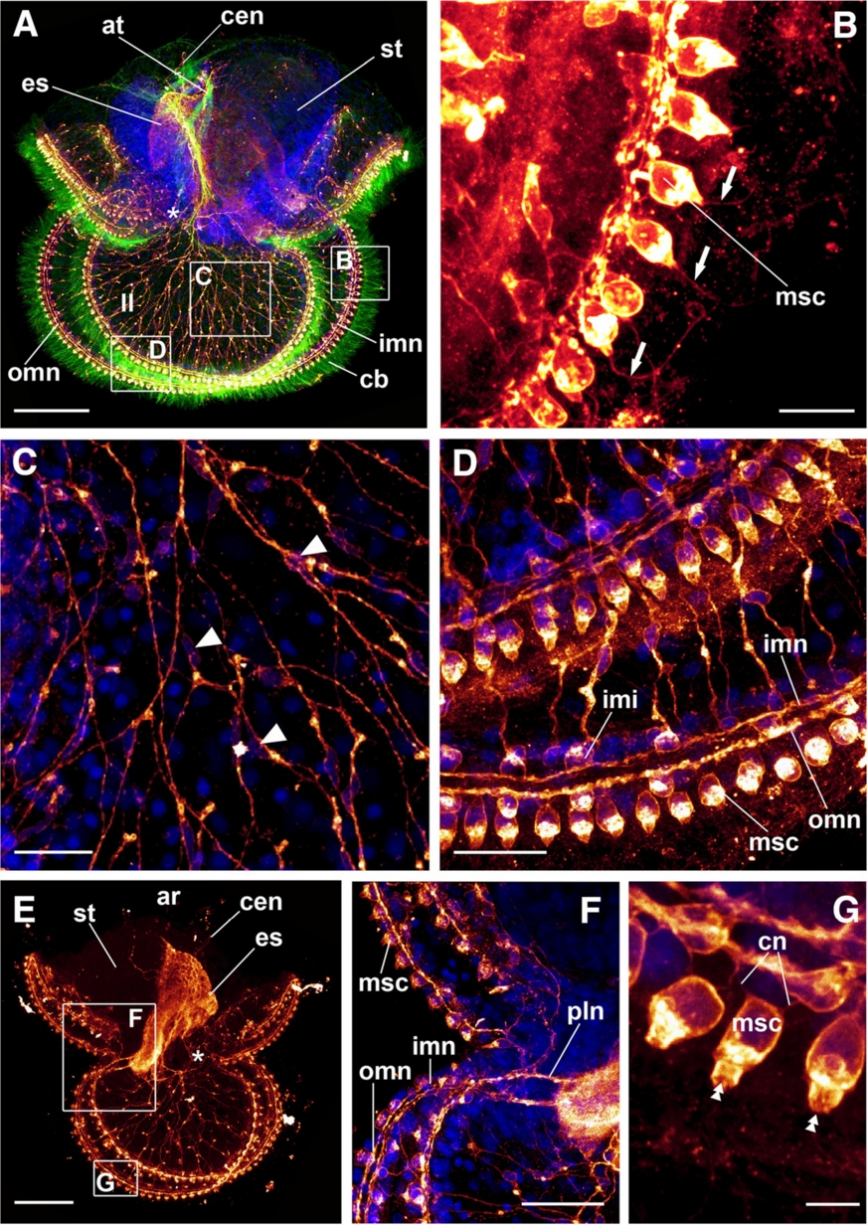
**FMRFamide-lir structures (graded scales of dark red to bright yellow) in *Lineus albocinctus *larvae.** Cell nuclei in blue, cilia in green. Boxes indicate regions of the respective detailed micrographs, which partly stem from different individuals. Apical faces upwards. Images are maximum projections of scans through the entire specimen. (**A**) A circumesophageal neurite proceeds from the apical part of the esophagus to its distal end. An outer marginal neurite bundle is present underneath the ciliary band. Lateral lobes show an additional inner marginal neurite bundle. Scale bar 100 μm. (**B**) Posterior lobe, detail. Marginal sensory cells are associated with the outer marginal neurite bundle. Scale bar 10 μm. (**C**) Lobar nerve net, detail. Scale bar 20 μm. (**D**) Detail of the inner and outer marginal neurite bundle of the lateral lobe. Inner marginal interneurons connect the processes of the lobar nerve net to the inner marginal neurite bundle. Scale bar 20 μm. (**E**) Larva with incorporated trunk disc stage. The circumesophageal neurite splits and its descendants form a complex lobar nerve net. Scale bar 60 μm. (**F**) Transition between the posterior and the lateral lobes, detail. The circumesophageal neurite splits into several peripheral lobar neurites, of which one on each side forms the inner marginal neurite bundle. Scale bar 30 μm. (**G**) Marginal sensory cells, detail. Two neurites connect the marginal sensory cells to the outer marginal neurite bundle. Scale bar 5 μm. Abbreviations: *, mouth; ar, apical region; at, apical ciliary tuft; cb, ciliary band; cen, circumesophageal neurite; cn connective neurite; es, esophagus; imi, inner marginal interneuron; imn, inner marginal neurite bundle; ll, lateral lobe; omn, outer marginal neurite bundle; pln, peripheral lobar neurite; msc, marginal sensory cell; st, stomach; arrows, single cilium; arrowheads, cell soma of multipolar interneuron; double arrowheads, microvilli collar.

**Figure 6:**
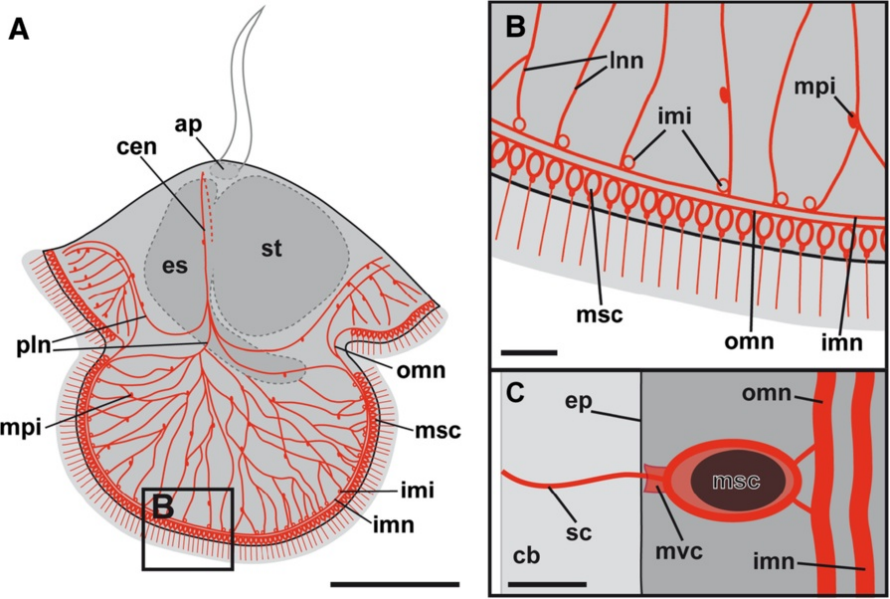
**Line drawings showing the larval FMRFamide-lir nervous system of *Lineus albocinctus*.** FMRFamide-lir structures are in red. Apical faces upwards. (**A**) Larva with circumesophageal neurite that descends on each side of the esophagus. At the level of the lobar junctions it splits into several peripheral lobar neurites. In the lateral lobes some of these peripheral lobar neurites form a complex nerve net with numerous interconnected multipolar interneurons. The neurites of the lateral lobar net are connected to the inner marginal interneurons, which are associated with the inner marginal neurite bundle. The inner marginal neurite bundle is only present in the lateral lobes and originates from one peripheral lobar neurite on each side of the lobe. An outer marginal neurite bundle underlies all four lobes and is associated with the marginal sensory cells. Scale bar 100 μm. (**B**) Detail of the distal part of the lateral lobe. The lateral nerve net is connected to the inner marginal neurite bundle via the inner marginal interneurons. The lobar nerve net shows multipolar interneurons which interconnect the neurites. The inner marginal neurite bundle is only present in the lateral lobes. Scale bar 20 μm. (**C**) Detail of a marginal sensory cell of the lateral lobe. Its single sensory cilium projects into the ciliary band and is surrounded by a microvilli collar. The marginal sensory cell is connected to the outer marginal neurite bundle via two neurites. Scale bar 5 μm. Abbreviations: ap, apical plate; cb, ciliary band; cen, circumesophageal neurite; ep, epidermis; es, esophagus; imi, inner marginal interneuron; imn, inner marginal neurite bundle; lnn, lateral nerve net; mpi, multipolar interneuron; msc, marginal sensory cell; mvc, microvilli collar; omn, outer marginal neurite bundle; pln, peripheral lobar neurite; sc, sensory cilium; st, stomach.

In larvae with incorporated trunk discs all four larval lobes are underlain by an outer marginal neurite bundle. The lateral lobes exhibit an additional inner marginal neurite bundle (Figures 5A, D and 6A, B). Along either side of the larval esophagus a circumesophageal neurite with few associated FMRFamide-lir cells is present (Figures 5E and 6A). At the height of the lobe junctions the circumesophageal neurite gives rise to several peripheral lobar neurites (Figures 5A, E and 6A). Parts of these peripheral lobar neurites and their descendants form a complex nerve net that contains numerous multipolar interneurons in the lateral lobes (Figure 5C). The neurites extend into the periphery of the lobes, where they are connected to the FMRFamide-lir inner marginal neurite bundle via the FMRFamide-lir inner marginal interneurons (Figures 5A, D, E and 6B). Two of the peripheral lobar neurites merge with the inner marginal neurite bundle on either side of the lobe at the lobe junctions (Figures 5F and 6A). In addition, a complex nerve net with several interconnected FMRFamide-lir interneurons is found in the anterior and posterior lobe (Figure 6A). In every lobe it consists of parts of the outer marginal neurite bundle and a strand of the peripheral lobar neurites (Figures 5F and 6A). In all four lobes distinct FMRFamide-lir cells are connected to the outer marginal neurite bundle by two neurites (Figures 5G and 6C). These cells are termed “marginal sensory cells” herein and bear a long, single cilium surrounded by a collar of microvilli (Figures 5B, G and 6B, C). These marginal sensory cells constitute a new type of larval sensory cell for Nemertea that is described for the first time herein. Remarkably, neither of these cells, nor any of the inner marginal interneurons, are present at the transition between individual lobes (Figures 5F and 6A). In the apical region no FMRFamide-lir structures were found in any of the larval stages investigated (Figures 5E and 6A).

### VD1/RPD2 α-neuropeptide-lir structures in the early pilidium larva

**Figure 7:**
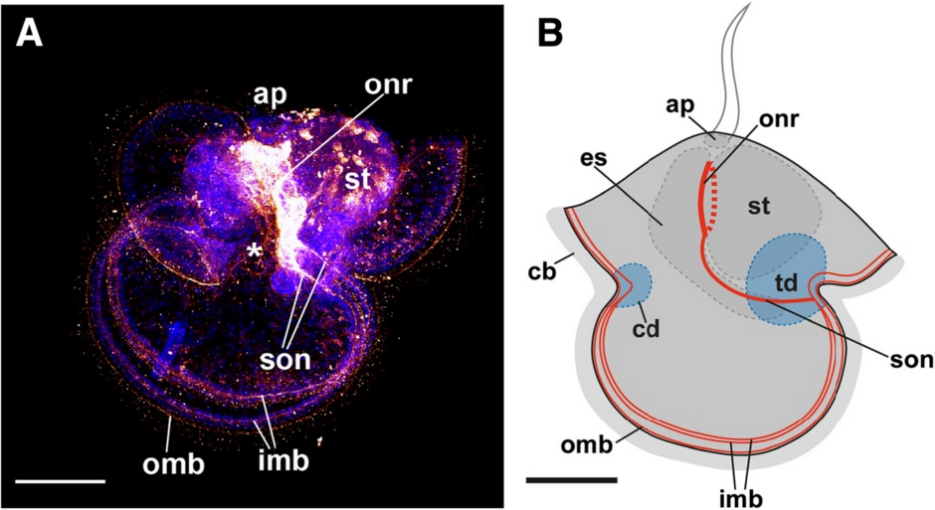
**VD1/RPD2 α-neuropeptide-lir structures in an early larva of *Lineus albocinctus*.** Apical faces upwards in both aspects. (**A**) Maximum projection of a scan through the entire specimen VD1/RPD2 α-neuropeptide-lir is shown in graded scales of dark red to bright yellow, cell nuclei are shown in blue. The oral nerve ring encircles the junction between the esophagus and the stomach. Two suboral neurites descend from the oral nerve ring towards the transition between the posterior and the lateral lobes. The anterior and the posterior lobes are underlain by two marginal neurite bundles. The inner marginal neurite bundle splits into two separate neurite bundles at the transition of the lobes. (**B**) VD1/RPD2 α-neuropeptide-lir structures are shown in red. All scale bars equal 100 μm. Abbreviations: *, mouth; ap, apical plate; cb, ciliary band; cd, cephalic disc; es, esophagus; imb, inner marginal neurite bundle; omb, outer marginal neurite bundle; onr, oral nerve ring; son, suboral neurite; st, stomach; td, trunk disc.

In larvae with incorporated trunk disc stages a positive VD1/RPD2 α-neuropeptide-lir signal along the oral nerve ring and the suboral neurites is present. The suboral neurites join the inner marginal neurite bundle, which is also stained, at the transition between the posterior and the lateral lobes (Figure 7A, B). In the lateral lobes the inner marginal neurite bundle splits into two neurite bundles, which are situated closely adjacent to the FMRFamide-lir inner and outer marginal neurite bundles. Whether or not these neurite bundles are identical remains unknown, because we were not able to perform reliable co-localization experiments, since both primary antibodies stemmed from the same host species (rabbit). An additional outer marginal neurite runs along the four lobes at the very distal border of the epidermis (Figure 7A, B). The signal found in the anterior region of some individuals (see Figure 7A) was due to unspecific staining and does therefore not show neuronal subsets.

## Discussion

### Development of the serotonin-lir nervous system in larval and juvenile nemerteans

Recently, two serotonin-lir apical cells as well as two serotonin-lir cells which lie beneath the apical cells were described for the hoplonemertean *Quasitetrastemma stimpsoni*. These cells are all flask-shaped and most likely homologous to similar cells found in the apical organ of many lophotrochozoan larvae [21,22]. In Pilidiophora, neurogenesis has been investigated in several species using TEM and immunocytochemical staining. We found that a serotonin-lir marginal neurite bundle that underlies the ciliary band is present in all four lobes of *Lineus albocinctus*, which corroborates previous studies on the same species as well as on *Micrura alaskensis*[9,16]. The marginal neurite bundle in *Lineus albocinctus* was reported to show numerous associated serotonin-lir cells which form contact with the marginal neurite bundle via two processes [16]. These associated serotonin-lir cells in *Lineus albocinctus* were also found in our study, while a connection to the marginal neurite bundle via two processes could not be confirmed. Interestingly, in *Lineus albocinctus*, every serotonin-lir cell associated with the marginal neurite bundle bears a single cilium. These findings on serotonin-lir structures are partly in accordance with data on *Micrura alaskensis*, which likewise exhibits a marginal neurite bundle with numerous associated serotonin-lir cells [9]. However, the latter work provides no information about the innervation of these associated serotonin-lir cells, nor does it report any ciliary structures that project from these cells.

During larval development of *Lineus albocinctus* the number of serotonin-lir cells associated with the marginal neurite bundle increases. The previously described serotonin-lir subepithelial nerve net with its interconnected multipolar interneurons as well as the increase in complexity of the nerve net during subsequent development is confirmed herein (see [9,16]). A serotonin-lir oral nerve ring has been described for *Lineus albocinctus* and *Micrura alaskensis*, albeit depiction is lacking for the latter species [9,16]. So far, the presence of two monociliated serotonin-lir apical neurons had been demonstrated for *Micrura alaskensis* only [9]. In our study, two serotonin-lir apical neurons are shown in the apical plate of *Lineus albocinctus* for the first time, although they are not flask-shaped, thus rendering homology to the apical cells reported for the hoplonemertean *Quasitetrastemma* (see above) and other, apical organ-bearing lophotrochozoans, unlikely.

The apical neurons of *Lineus albocinctus* send their processes into each of the four lobes. An apical neurite plexus that underlies the apical plate is formed by the processes of the apical neurons. These findings contradict those of the previous study, where no neural structures in the apical plate of *Lineus albocinctus* were found [16].

During postlarval development of *Lineus albocinctus* the serotonin-lir oral nerve ring and the serotonin-lir suboral neurite are incorporated into the juvenile serotonin-lir nervous system. This and the fusion of the suboral neurites with the lateral neurite bundles of the juvenile clearly demonstrates that larval structures form parts of the juvenile nervous system. Confirming an earlier study on *Lineus albocinctus*, two serotonin-lir lateral neurite bundles are present in the ventro-lateral region of the juvenile worm [16]. In *Micrura alaskensis* two neural structures have been interpreted as lateral neurite bundles of the juvenile, but these structures were identified by phalloidin, a stain specific for filamentous actin rather than defined neural structures [9]. The cell progenies of the cephalic discs and the cerebral organ discs of *Micrura alaskensis* have been proposed to contribute to the development of the lateral neurite bundles [9]. This is confirmed by the results of our study, although it has to be mentioned that former methodical approaches by use of phalloidin for visualizing neural, in particular serotonin-lir structures [9], renders these previous data doubtful. As in *Micrura alaskensis*[9], longitudinal serotonin-lir neurites run through the proboscis *anlage* of *Lineus albocinctus*.

### Development of the FMRFamide-lir neural structures in larval and juvenile nemerteans

The data presented herein show a much higher grade of complexity of the larval FMRFamide-lir system in *Lineus albocinctus* than reported by an earlier study [16]. A circumesophageal neurite loops around the apical part of the esophagus and descends into the abapical region of the episphere. The circumesophageal neurite is associated with few FMRFamide-lir cell bodies. Due to its position within the larva and its FMRFamide-like immunoreactivity, this circumesophageal neurite may constitute the very process previously termed “lateral helmet process” [16]. The present study shows that this neurite splits into several peripheral lobar neurites in the region of the lobe junctions in the *Lineus albocintus* pilidium. A complex lobar nerve net with numerous multipolar interconnected interneurons originates from the peripheral lobar neurites. In addition, the peripheral lobar neurites contribute to the inner marginal neurite bundle of the lateral lobes. The neurites of the lobar nerve net are always connected to the inner marginal neurite bundle of the lateral lobes via inner marginal interneurons. An outer marginal neurite bundle, which is associated with FMRFamide-lir marginal sensory cells, runs along the four lobes. Due to the presence of a single cilium, the microvilli collar and the connection to the marginal neurite bundle, these cells may correspond to a cell type shown previously by TEM studies of an unknown pilidiophoran species [8].

Throughout the different developmental stages investigated no essential changes in the FMRFamide-lir nervous system of the larva were found. No FMRFamide-lir structures were found in the region of the apical plate.

## Conclusions

Recently, an apical organ that includes four flask-shaped serotonin-lir apical neurons was found in the hoplonemertean *Quasitetrastemma stimpsoni*[21]. This was the first work that unequivocally showed the existence of an apical organ that contains distinct sensory cells in any nemertean larva and suggests that such a morphological feature may have been part of the hoplonemertean groundplan. In Pilidiophora, the serotonin-lir apical neurons in *Micrura alaskensis* show a single cilium, but are not flask-shaped and lack the connection to the apical cilia, and those in *Lineus albocinctus* are neither flask-shaped, nor do they appear to bear a single cilium. Thus, these cells are not indicative of a rudimentary apical organ. Since many lophotrochozoans, such as Mollusca, polychaete annelids, Phoronida and Entoprocta, exhibit an apical organ, it is more parsimonious to assume that such a structure was also present in the LCA of Lophotrochozoa, and was secondarily lost in several subclades, including Pilidiophora. Our data also demonstrate the previously overlooked high complexity of the larval FMRFamide-lir nervous system in *Lineus albocinctus* and adds the mollusk-specific VD1/RPD2 α-neuropeptide to the suite of nemertean (larval) neural components. This finding calls for further investigations into other lophotrochozoan and bilaterian taxa to assess its degree of conservation in the respective clades. Accordingly, large-scale comparative immunocytochemical studies using the antibody against this peptide could reveal an important set of novel data that may prove highly useful for (neuro)phylogenetic studies.

The oral nerve ring and the two suboral neurites are incorporated into the juvenile nervous system of *Lineus albocinctus*. The two lateral neurite bundles develop inside the juvenile imaginal discs and most likely form the future ventral nerve cords of the adult nemertean. Based on comparative juvenile and adult neuroanatomy, it appears most likely that the incorporated oral nerve ring develops into (parts of) the adult brain commissures. This requires, however, that the juvenile mouth develops secondarily, because only then the oral nerve ring would encircle the proboscis but not the esophagus, corresponding to the situation found in adult Nemertea. This scenario would also provide an explanation for the formation of the adult brain by concentration (fusion?) of the oral nerve ring and the anterior connection of the lateral neurite bundles. However, data on neurogenesis from early juveniles through adulthood are needed to further assess this issue.

## Materials and methods

### Animal collection, determination and fixation

In pilidium larvae of *Lineus albocinctus* the anterior lobe is more prominent than the posterior one. The helmet as well as the anterior and the posterior lobes show a concave outline. The apical tuft can be as long as the entire height of the larva, and the apical plate, formed by epidermal cells, is relatively large.

Larvae of different developmental stages were collected in August 2011 in the Gullmarsfjord, Swedish west coast by plankton tows (58°15′7″N, 11°26′30″E; 58°15′02″N, 11° 27′23″E). Animals were relaxed in a 3.5% MgCl_2_ solution and were fixed for approximately 1.5 to 2 hours at room temperature (RT) in 4% paraformaldehyde in 0.1 M PBS (phosphate buffered saline, pH 7.4) with 10% sucrose added. Afterwards, animals were rinsed in 0.1 M PBS with 0.1% NaN_3_ and stored in the same solution at 4°C.

### Immunocytochemistry and confocal laserscanning microscopy

For confocal microscopy larval tissue was permeabilized for 60 min in PBT (0.1 M PBS with 0.1% NaN_3_ and 4% Triton X-100). Non-specific binding sites were blocked in PBT with 6% normal goat serum (Jackson ImmunoResearch, West Grove, PA, USA) at RT overnight. Subsequently, the larvae were incubated in either of the following primary antibodies: 5-HT antibody (serotonin) raised in rabbit, diluted 1:800 (ImmunoStar, Hudson, WI, USA); FMRFamide antibody raised in rabbit, diluted 1:500 (Biotrend, Cologne, Germany); a mollusk-specific α-neuropeptide antibody, diluted 1:350 (CASLO ApS, Lyngby, Denmark), directed against the VD1/RPD2 system of the pond snail *Lymnaea stagnalis*[17] raised in rabbit; acetylated-α-tubulin antibody raised in mouse, diluted 1:400 (Sigma-Aldrich, St. Louis, MO, USA) with blockPBT (PBT with 6% goat serum) for 24 hours at RT. Specimens were rinsed four times in blockPBT for at least 6 hours. Then, they were incubated for 24 hours at RT in Alexa Fluor 568 anti-rabbit (Invitrogen, Molecular Probes, Eugene, OR, USA) or Alexa Fluor 633 anti-mouse (Invitrogen) secondary antibody, respectively, both at a dilution of 1:300 in blockPBT. DAPI (Sigma) was added at a 1:400 dilution for staining of the cell nuclei. Stained larvae were washed four times in PBS for at least six hours and then mounted in Fluoromount-G (Southern Biotech, Birmingham, AL, USA). Cover glasses were provided with clay feet to prevent squashing of specimens. Slides were stored at 4°C.

Samples were examined with a Leica SP5 II confocal microscope (Leica Microsystems, Wetzlar, Germany) and stacks of optical sections of 0.3-0.6 μm Z-thickness were generated. Further digital image processing was done with Adobe Photoshop CS (Adobe, San Jose, CA, USA). Adobe Illustrator CS (Adobe) was used for creating the line drawings.

## Competing interests

The authors declare that they have no competing interests.

## Authors’ contributions

SH generated all data, analysed the results, and drafted the manuscript. AW designed and supervised research, contributed to data interpretation, and finalized the manuscript. TS contributed to obtaining the study material and confocal microscopy analysis. All authors read and approved the final version of the manuscript.
